# Supplementary material for: Modeling glioblastoma heterogeneity as a dynamic network of cell states
Source: Mol Syst Biol. 2021 Sep 16;17(9):e10105. doi: 10.15252/msb.202010105 (PMC8444284; doi:10.15252/msb.202010105)
Supplement: Supplementary file 5 — Source Data for Figure 3 [file MSB-17-e10105-s001.zip › Figure3A_sourcedata/GSEA_3065/hallmarks_state2.GseaPreranked.1623416269049/HALLMARK_REACTIVE_OXYGEN_SPECIES_PATHWAY.html]

Details for gene set HALLMARK\_REACTIVE\_OXYGEN\_SPECIES\_PATHWAY[GSEA]

|  || Dataset | state2 |
| Phenotype | NoPhenotypeAvailable |
| Upregulated in class | na\_pos |
| GeneSet | HALLMARK\_REACTIVE\_OXYGEN\_SPECIES\_PATHWAY |
| Enrichment Score (ES) | 0.38226274 |
| Normalized Enrichment Score (NES) | 1.0900832 |
| Nominal p-value | 0.3353846 |
| FDR q-value | 0.39939597 |
| FWER p-Value | 1.0 |
Table: GSEA Results Summary

  

Fig 1: Enrichment plot: HALLMARK\_REACTIVE\_OXYGEN\_SPECIES\_PATHWAY      
 Profile of the Running ES Score & Positions of GeneSet Members on the Rank Ordered List

  

| PROBE | GENE SYMBOL | GENE\_TITLE | RANK IN GENE LIST | RANK METRIC SCORE | RUNNING ES | CORE ENRICHMENT || 1 | NDUFA6 |  |  | 338 | 0.222 | 0.0440 | Yes |
| 2 | GPX4 |  |  | 425 | 0.194 | 0.1047 | Yes |
| 3 | PDLIM1 |  |  | 452 | 0.190 | 0.1705 | Yes |
| 4 | CDKN2D |  |  | 499 | 0.182 | 0.2310 | Yes |
| 5 | PRDX2 |  |  | 990 | 0.121 | 0.2224 | Yes |
| 6 | NDUFB4 |  |  | 1027 | 0.117 | 0.2609 | Yes |
| 7 | SOD1 |  |  | 1028 | 0.117 | 0.3032 | Yes |
| 8 | ATOX1 |  |  | 1246 | 0.102 | 0.3168 | Yes |
| 9 | GLRX2 |  |  | 1313 | 0.097 | 0.3448 | Yes |
| 10 | LAMTOR5 |  |  | 1499 | 0.087 | 0.3564 | Yes |
| 11 | GSR |  |  | 1739 | 0.075 | 0.3580 | Yes |
| 12 | PRDX1 |  |  | 1763 | 0.074 | 0.3823 | Yes |
| 13 | HMOX2 |  |  | 3104 | 0.032 | 0.2511 | No |
| 14 | PFKP |  |  | 3170 | 0.030 | 0.2551 | No |
| 15 | TXN |  |  | 3221 | 0.029 | 0.2603 | No |
| 16 | SOD2 |  |  | 3366 | 0.026 | 0.2546 | No |
| 17 | NDUFS2 |  |  | 3438 | 0.025 | 0.2561 | No |
| 18 | STK25 |  |  | 3490 | 0.024 | 0.2593 | No |
| 19 | PRDX6 |  |  | 3639 | 0.022 | 0.2513 | No |
| 20 | JUNB |  |  | 3923 | 0.017 | 0.2273 | No |
| 21 | GCLC |  |  | 4056 | 0.015 | 0.2185 | No |
| 22 | MSRA |  |  | 4178 | 0.013 | 0.2102 | No |
| 23 | GCLM |  |  | 4866 | 0.002 | 0.1377 | No |
| 24 | PTPA |  |  | 5228 | -0.004 | 0.1006 | No |
| 25 | G6PD |  |  | 5598 | -0.010 | 0.0650 | No |
| 26 | SBNO2 |  |  | 6107 | -0.018 | 0.0175 | No |
| 27 | OXSR1 |  |  | 6234 | -0.021 | 0.0115 | No |
| 28 | TXNRD2 |  |  | 6294 | -0.022 | 0.0130 | No |
| 29 | TXNRD1 |  |  | 6419 | -0.024 | 0.0085 | No |
| 30 | ERCC2 |  |  | 6652 | -0.028 | -0.0060 | No |
| 31 | SCAF4 |  |  | 6740 | -0.030 | -0.0043 | No |
| 32 | CAT |  |  | 7094 | -0.039 | -0.0279 | No |
| 33 | FTL |  |  | 7693 | -0.056 | -0.0712 | No |
| 34 | GLRX |  |  | 8100 | -0.071 | -0.0887 | No |
| 35 | NQO1 |  |  | 8837 | -0.127 | -0.1211 | No |
| 36 | PRNP |  |  | 9068 | -0.161 | -0.0876 | No |
| 37 | SELENOS |  |  | 9135 | -0.174 | -0.0318 | No |
| 38 | PRDX4 |  |  | 9151 | -0.178 | 0.0307 | No |
Table: GSEA details [plain text format]

  

Fig 2: HALLMARK\_REACTIVE\_OXYGEN\_SPECIES\_PATHWAY: Random ES distribution      
 Gene set null distribution of ES for **HALLMARK\_REACTIVE\_OXYGEN\_SPECIES\_PATHWAY**
